# The effect of lower inter-limb asymmetries on athletic performance: A systematic review and meta-analysis

**DOI:** 10.1371/journal.pone.0286942

**Published:** 2023-06-08

**Authors:** Kai T. Fox, Liam T. Pearson, Kirsty M. Hicks

**Affiliations:** affiliations>Department of Sport, Exercise and Rehabilitation; Northumbria University, Newcastle Upon Tyne, United Kingdom; Instituto Politecnico de Viana do Castelo, PORTUGAL

## Abstract

Inter-limb asymmetry refers to an imbalance in performance between the left and right limbs. Discrepancies throughout asymmetry research does not allow practitioners to confidently understand the effect of inter-limb asymmetries on athletic performance. Therefore, this review summarized the current literature using a meta-analytic approach, conforming to the Preferred Reporting Items for Systematic Review and Meta-Analyses guidelines to identify the association between inter-limb asymmetry and athletic performance. A literature search using PubMed, Web of Science and SPORTDiscus databases yielded 11-studies assessing the effect of inter-limb asymmetries, measured via unilateral jump performance, on bilateral jump, change of direction (COD) and sprint performance in adult sports players. The quality of evidence was assessed via a modified Downs and Black checklist and in compliance with the Grading of Recommendations Assessment Development and Evaluation. Correlation coefficients were transformed via Fishers z (*Z*_*r*_), meta-analysed and then re-converted to correlation coefficients. Egger’s regression presented no significant risk of bias. Vertical jump performance was not significantly affected by asymmetry (*Z*_*r*_ = 0.053, *r* = 0.05; *P* = 0.874), whereas COD and sprint both presented significant weak associations (COD, *Z*_*r*_ = 0.243, *r* = 0.24; Sprint, *Z*_*r*_ = 0.203, *r* = 0.2; *P* < 0.01). The results demonstrate that inter-limb asymmetries seem to present a negative impact to COD and sprint performance but not vertical jump performance. Practitioners should consider implementing monitoring strategies to identify, monitor and possibly address inter-limb asymmetries, specifically for performance tests underpinned by unilateral movements such as COD and sprint performance.

## 1.0. Introduction

Inter-limb asymmetry refers to imbalances in performance between opposing limbs [1, 2]. Associations between unilateral and bilateral performance [3, 4], have led to investigations of whether decremented single-limb ability exists in bilateral exercises, hindering athletic performance [5–9]. The development of inter-limb asymmetries have been attributed to several contributors such as; sports that incorporate repetitive asymmetrical movements [10–13], < 8-years training age (likely due to diminishing returns in the dominant limb as training age increases) [10, 14, 15], injuries, and anatomical asymmetries [16–18]. Interestingly, a recent meta-analysis found no association between lateral preference (preferred choice of limb to complete unilateral motor tasks) and inter-limb asymmetry [19], although previously been acclaimed as a contributing factor for the prevalence of inter-limb asymmetry, identifying the pre-existing complexity and controversy surrounding the research area [20]. Typically, due to sporting movements and physical qualities associated with sporting performance being underpinned by lower limb musculature, research has predominately focused on the effect of inter-lower-limb asymmetries on performance [6, 8, 13, 21–25]. Jumping, change of direction (COD), and sprint ability are regularly selected as performance tests within the literature due to their association with on-field performance (*r* = 0.12–0.75) [7, 9], and their use for talent identification [26–28]. Thus, if inter-limb asymmetries within the lower limbs were to impair these performance measures, it could impact an athlete’s or team’s sporting performance. Hence, understanding the effects of inter-limb asymmetries on vertical jump, COD and sprint ability is essential to inform practitioners on whether monitoring strategies and training interventions are necessary to optimise athletic performance.

Whether inter-limb asymmetries translate to an effect on athletic performance remains unclear, with studies supporting [1, 6, 7, 9, 21], or refuting [8, 25, 29–32], an effect. For example, Loturco et al. [8] found that 11% asymmetries in unilateral countermovement jumps (UCMJ) were not associated with jump, COD or sprint performance in elite women. They suggested that elite athletes might have neuromechanical qualities that compensate for the potential decrements in bilateral performance due to asymmetries [8]. In agreement with this suggestion, computer simulations have demonstrated the ability to alter movement mechanics in order to maintain performance when asymmetries were present [33]. Controversially, Bishop et al. [6] found unilateral drop jump asymmetries (UDJ) to significantly correlate to decrements in sprint and COD performance, suggesting that not all asymmetries can be compensated for by altering movement. The discrepancies between the Loturco et al. [8] and Bishop et al. [6] results could be attributed to the different asymmetry assessments used and the varying level of assessment difficulty (i.e., UCMJ versus UDJ). It is well-known that asymmetry is task-specific, with literature stating that multiple measures should be done to assess asymmetry [30, 34], thus the single measurements used within the aforementioned studies present potential inaccuracy in the asymmetries found which could have skewed the correlations presented.

Despite the potential impact of asymmetries, there remains discrepancies in asymmetry research highlighting the lack of clarity for practitioners to understand the true effects of asymmetry on performance and the necessity for further research. Thus, the aim of this study was to systematically review and meta-analyse the available studies on the effect of inter-limb asymmetries on jump, COD, and sprint performance in sporting cohorts. A meta-analysis was conducted to summarise and statistically quantify the data from individual studies, to increase the statistical power of the study [35].

## 2.0. Methods

### 2.1. Design

This review conformed to the Preferred Reporting Items for Systematic Reviews and Meta-analyses (PRISMA) 2020 guidelines [36]. The following steps (establishing the inclusion-exclusion criteria, literature search, literature selection, quality appraisal and data extraction) were employed by independent reviewers (KTF and LTP) and controversies were resolved via discussion with the third reviewer (KMH). The study aims and design were ethically approved prior to any experimental searches. The study aims and design were ethically approved in compliance with the Northumbria Universities Faculty of Health and Life Sciences governance regulations prior to any experimental searches with written informed consent (Ethics: Reference number 33257).

### 2.2. Inclusion-exclusion criteria

Literature was screened in line with the inclusion-exclusion criteria below. Population, Comparator, Outcome and Study design was used to establish the parameters of which this review was conducted in accordance with PICOS.

#### 2.2.1. Population

Studies included within the review used participants that were healthy men and women who were: aged 18–35 years; participated in a sport involving jumping, COD and/or sprinting, free from any injury or disability, and had not undergone surgery that may cause inter-limb asymmetry. No restrictions were given for training age or level.

#### 2.2.2. Comparator

Studies that were included compared the associations between inter-limb asymmetry and jump, COD, and/or sprint performance between participants. All studies quantified asymmetry within the lower limbs via UCMJ, UDJ and/or unilateral squat jump (USJ) height, as these provide implications into asymmetrical physical qualities such as the stretch shortening cycle that underpin athletic performance and remain accessible and inexpensive [37].

#### 2.2.3. Outcomes

The primary outcome was to test the effect of inter-limb asymmetry on athletic performance. For the purpose of this review, athletic performance was defined as an individual’s jump, COD and sprint performance. All data were categorized into three individual performance measures; bilateral jumping (countermovement jump (CMJ), drop jump (DJ) and/or squat jump (SJ)), COD (no limit on the number of cuts/turns), and sprint performance.

#### 2.2.4. Study design

No specific study design was needed to be included within this review. Studies were considered for inclusion if they were: published in full, in a peer-reviewed journal; written in English or had a previously accessible translation to be retrieved; and had the primary or secondary aim to assess the effect of lower, inter-limb asymmetries on athletic performance. Dissertations, abstracts, and conference papers were excluded.

### 2.3. Literature search

A systematic literature search was conducted electronically by KTF, identifying all relevant articles using PubMed, Web of Science, and SPORTDiscus, from inception of the database until 22/03/2023. Search terms included keywords from relevant literature [2, 38] and used Boolean logic and truncations as follows: (‘Asymmetr*’) AND (‘lower limb’ OR ‘inter-limb’ OR ‘strength’ OR ‘muscle’ OR ‘unilateral’ OR ‘bilateral’ OR ‘dominant leg’ OR ‘non-dominant leg’ OR ‘jump*’) AND (‘performance’ OR ‘athletic performance’ OR ‘jump*’ OR ‘sprint*’ OR ‘change of direction’ OR ‘agility’) NOT (‘animals’ OR ‘adolescents’ OR ‘diseas*’ OR ‘disabl*’ OR ‘upper body’ OR ‘upper limbs’ OR ‘arms’ OR ‘Hands’ OR ‘injur*’ OR ‘gait’). Hand searches of reference lists were conducted on eligible literature and review articles, identifying any further studies that had been missed.

### 2.4. Study selection

**Fig 1:**
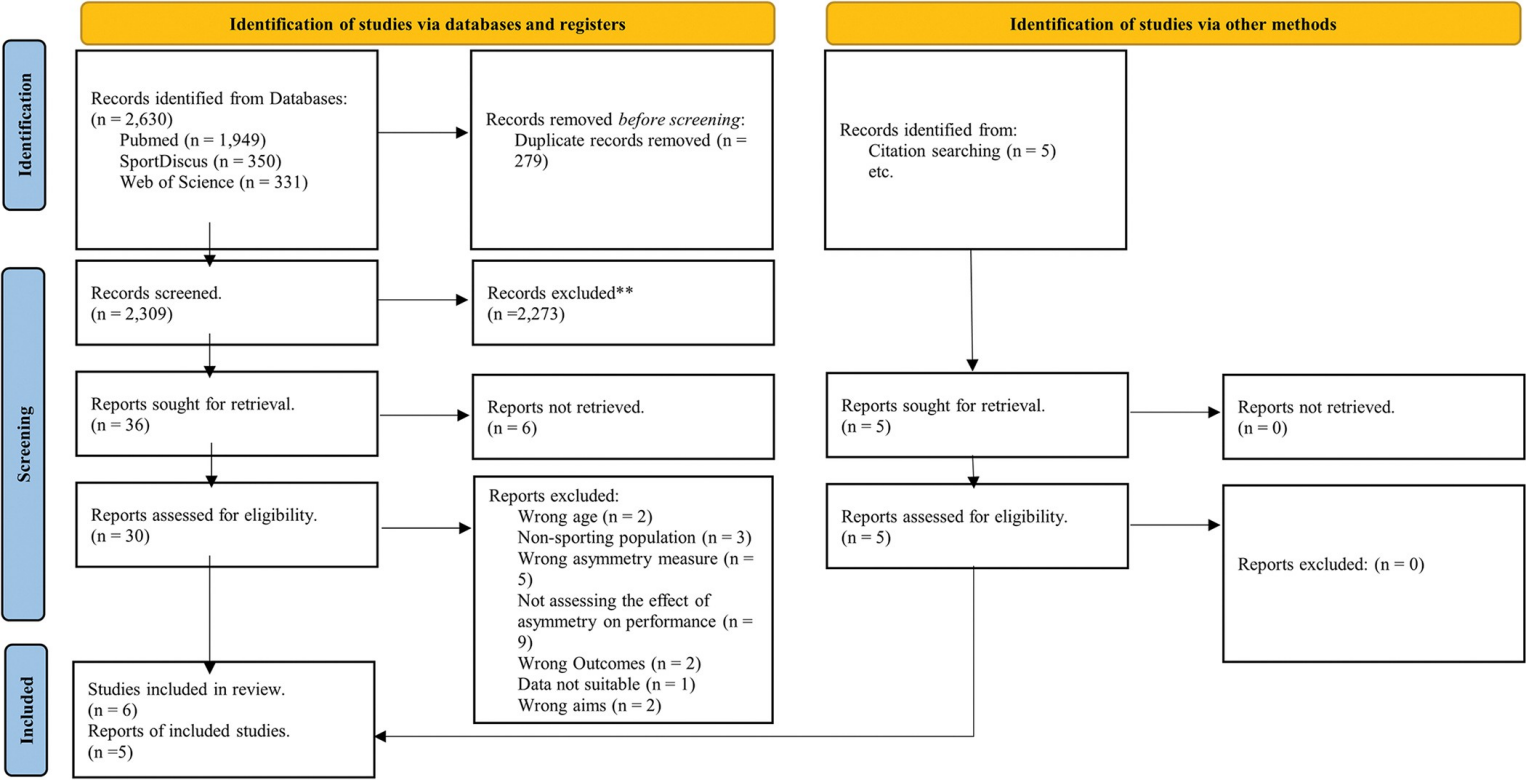
Preferred Reporting Items for Systematic Reviews and Meta-analyses (PRISMA) 2020 guidelines flow chart for literature search and inclusion.

Once duplicates were removed, two independent reviewers (KTF and LTP) conducted a two-stage screening process in line with the pre-determined inclusion-exclusion criteria (Fig 1), using the Rayyan web-based platform [39]. Stage one included screening the titles and abstracts for all database and hand reference searches. Stage two assessed the full-text manuscripts of all the remaining literature from stage one. Any study that did not meet the eligibility criteria or met at least one exclusion criterion at any stage was removed. Any conflicts between reviewers KTF and LTP were resolved by the third independent reviewer KMH.

### 2.5. Quality appraisal

Study quality was assessed independently by KTF and reviewed by LTP. Priori-study quality was determined via the Downs and Black [40] checklist modified to suit the current review. This comprised of 14-outcomes, with a total score of 15 been available, assessing the quality of literature on the individual studies reporting, external validity, internal validity-bias, internal validity-confounding, and power. Using guidelines set from previous research a score of 13–15 was deemed as ‘high’, 10–12 was ‘moderate’, 6–9 was ‘low’ and 0–5 was ‘very low quality’ [41]. The quality of evidence was then evaluated using the Grading of Recommendations Assessment and Evaluation (GRADE) criteria, initially assessing quality based on the study design, whereby the study quality was downgraded if using an observational approach. Quality was further assessed based on any of the following outcomes: risk of bias, indirectness, inconsistency, imprecision and publication bias [42]. Indirectness was evaluated, where the quality was maintained or downgraded depending on the answer to two questions that determined the directness within individual studies; Q1) was an accurate equation (the percentage difference or bilateral strength asymmetry (BSA) equation (Eqs 1. & 2.)) used to determine inter-limb asymmetry in line with Bishop et al. [43] and Q2) was the equipment used to identify unilateral jump height and subsequent outcome variables valid and reliable? The consistency of results was assessed via a visual interpretation of the transformed effect sizes. Publication bias was attempted to be avoided by using multiple databases and was assessed via Egger’s regression. The quality assessment was not used to include or exclude any studies.


PercentageDifference=100÷(maxvalue)×(minvalue)×(−1)+100 (1)

BSA=(BetterPerformingLimb−LesserPerformingLimb)÷BetterPerformingLimb×100 (2)

### 2.6. Data extraction

Data were extracted by KTF using a pre-piloted form, reviewed by LTP, to ensure all relevant study characteristics were extracted. If relevant data was limited, the authors were contacted and given six-weeks to respond and provide the appropriate material. If this information was not provided, the study was excluded from the meta-analysis. All raw data retrieved from the included studies and used for meta-analysis is available in the S1–S3 Appendices.

### 2.7. Statistical analyses

All data were extracted to an excel spreadsheet (Microsoft, 2018). All included studies employed observational designs, providing correlation coefficients. Data extracted across different performance measures or as different tests to assess the same performance measure (i.e., 20 m & 30 m sprint) within the same study was included as its own entity and underwent the same statistical process. One study assessed COD and sprint performance via m.s^-1^ compared to seconds; therefore, the correlation was reversed to fit the same correlation trendline for meta-analysis. Effect sizes were calculated using Fishers z (*Z*_*r*_) transformation where Zr and its standard error (*SEz*) were used for meta-analysis [44, 45]. The meta-analysis was conducted using JASP (JASP Team, 2020. Version 0.14.1), where the variation in effect, measured as *Z*_*r*_, was assumed to be random, hence the Hedges and Colleges random-effects method was employed. Heterogeneity was assessed via the *I*^*2*^ and Q-statistic where an *I*^*2*^ statistic output > 50% was considered a problematic amount of variability, in accordance with the *Cochrane Handbook for Systematic Reviews of Interventions* [46]. Forest plots provided a visual representation of the individual study effects and overall estimated mean effect (Figs 3–5). The omnibus test of model coefficients presented the significance of the effect, where a statistical significance was set at *P* < 0.05. The exact overall effect size and its 95% confidence intervals (95% CI) were identified and reported. Eggers regression test of funnel plot asymmetry was employed to assess small study bias (publication bias). The overall estimate effect sizes, expressed as *Z*_*r*_, were re-converted to a correlation coefficient providing the relationship between lower, inter-limb asymmetries and athletic performance. The magnitude of estimate effect was determined in line with Cohens *d* thresholds, whereby an effect size was categorised as trivial (< 0.2), small (0.2–0.5), moderate (0.5–0.8) and large (> 0.8) [47]. The strength of the association was interpreted in compliance with Evans [48], where the relationship (*r*-value) was considered very weak (≤ 0.19), weak (0.2–0.39), moderate (0.4–0.59), strong (0.6–0.79) or very strong (≥ 0.8).

## 3.0. Results

### 3.1. Study characteristics

The literature search and selection are presented in Fig 1. A total of 11 studies met the inclusion criteria for this review, consisting of 283 participants (men = 240; Women = 43), from a variety of sports, competing at a recreational to elite level [1, 6–9, 21, 25, 29–32]. All studies employed an observational design. One study assessed the effect of inter-limb asymmetries amongst multiple age groups [7], however data was only extracted from the over-18 age group in line with the pre-determined inclusion-exclusion criteria.

### 3.2. Quality of literature

**Fig 2:**
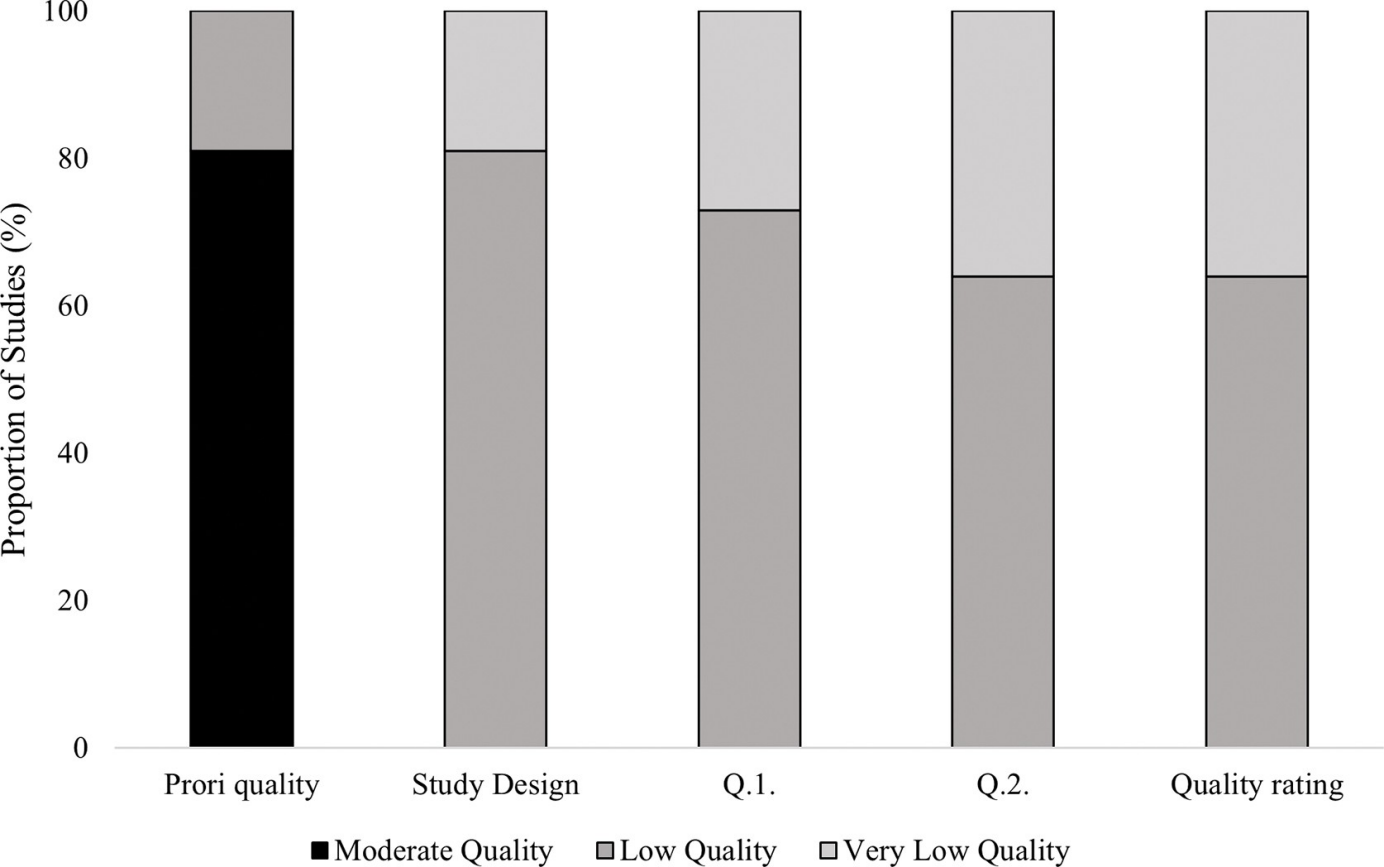
Quality of evidence for included studies (n = 11). Each bar represents the proportion of studies (%) rated as “Moderate” to “Very low” quality at each stage of the assessment, represented across the x-axis. The stages include the modified Downs and Black [40] appraisal score (priori-quality), GRADE criteria, assessing the study design, followed by two questions to assess study indirectness; Q.1. was an accurate equation used to determine inter-limb asymmetry? Q.2. Was the equipment used to identify unilateral jump height and subsequent outcome variables valid and reliable? The final bar signifies the final rating of evidence quality.

An assessment of quality for all included studies (*n* = 11) was conducted via a modified Downs and Black [40] appraisal tool and the GRADE criteria [42]. Quality assessment details are in Fig 2. A mean priori-quality score of 10.7 (moderate) was given, with all studies downgraded due to employing an observational design. Indirectness was presented in one study [29] on Q1 ‘was an accurate equation used to determine inter-limb asymmetry?’ and one study [9] on Q2 ‘was the equipment used to identify unilateral jump height and subsequent outcome variables valid and reliable?’, thus, these studies were further downgraded. Overall, the quality rating within this review includes four studies being “very low” and seven studies being “low” quality (Fig 2).

### 3.3. Jump performance

**Fig 3:**
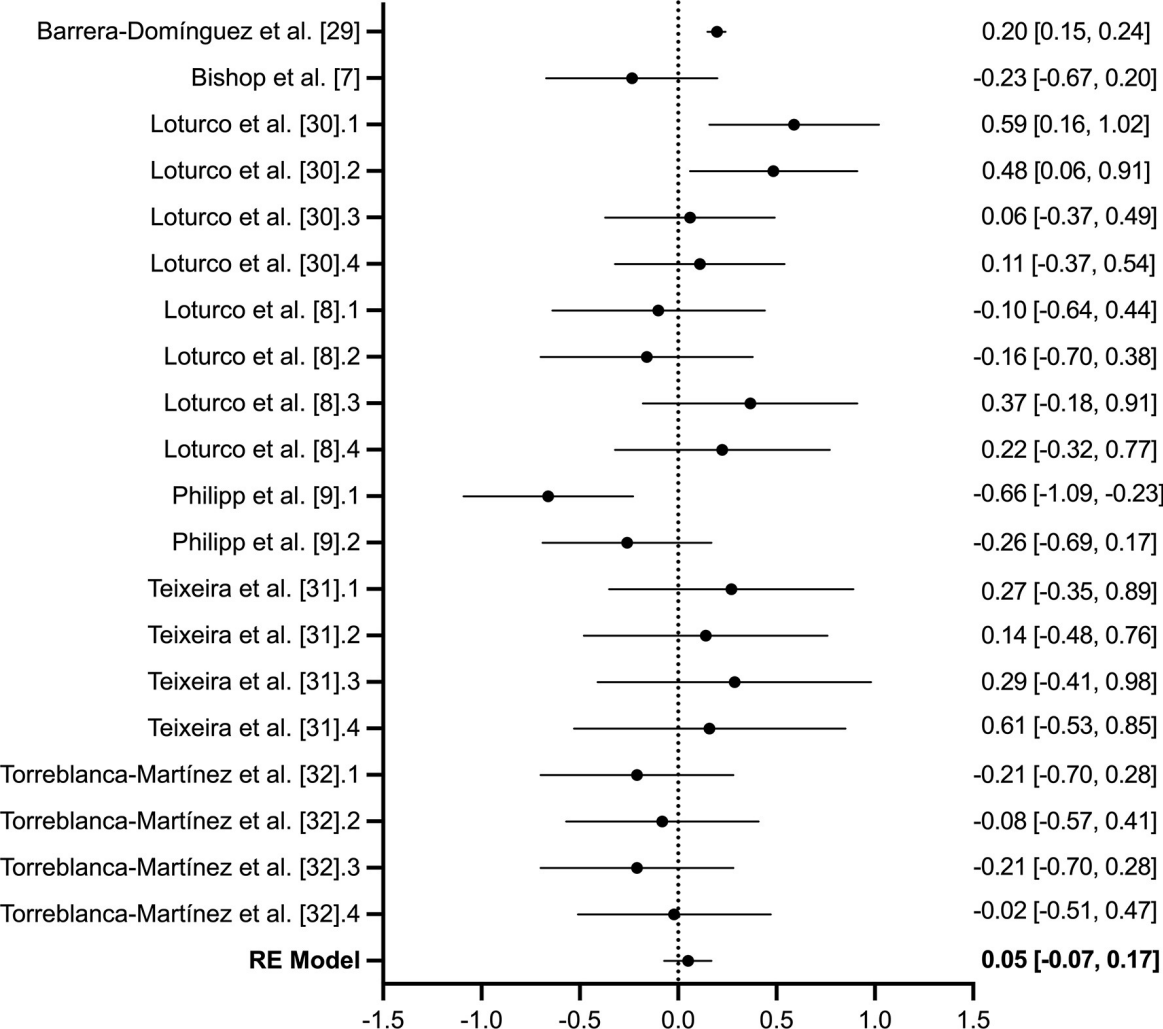
*Forest plot of the 20-transformed effect sizes across seven studies (*n *= 178) assessing the effect of inter-limb asymmetries on bilateral jump performance measured via countermovement*, *drop and/or squat jump*. The null line represents no correlation, with data presented to the left representing a negative correlation and to the right representing a positive correlation. For studies with repeated performance tests, a numerical order after the reference is used and aligns with the order presented in Table 1.

Seven studies (*n* = 178), of which 20-transformed effect sizes were provided, investigated the effect of inter-limb asymmetry on bilateral jump performance (Table 1). The quality of evidence assessing jump performance included three low quality studies and four very low-quality studies. Heterogeneity expressed as the *I*^*2*^ statistic was not substantial (*I*^*2*^ = 37.53%) although the Q-statistic was significant (*P* = 0.005). The overall estimate effect was insignificant (*Z*_*r*_ = 0.053 [95% CI: -0.066 to 0.173]; *r* = 0.05, [95% CI: -0.07 to 0.17]; *P* = 0.874; Fig 3). No significant bias was detected within eggers test (*P* = 0.567).

**Table 1:** Studies assessing the effect of lower, inter-limb asymmetries on bilateral jump performance (*n* = 130).

Study	Participants	Assessment of Asymmetry (Average Asymmetry)	Performance	Findings
Barrera-Domínguez et al. [29]	Competitive sport players (*n* = 48)	UDJ (13%)	CMJ	No effect (*r* = 0.194, *P* > 0.05)
Bishop et al. [7]	Elite soccer players (M; *n* = 23)	UCMJ (5.76%)	CMJ	No effect (*r* = -0.23, *P* > 0.05)
Loturco et al. [30]	Elite soccer players (M; *n* = 24)	UCMJ (7.9 ± 6.9%)	CMJ & SJ	No effect to CMJ (*r* = 0.53) and SJ (*r* = 0.45, *P* > 0.05)
		USJ (10.7 ± 6.6%)		No effect to CMJ (*r* = 0.06) and SJ (r = 0.11; both *P* > 0.05)
Loturco et al. [8]	Elite soccer players (F; *n* = 16)	USJ (9.8%)	SJ & CMJ	No effect to SJ (*r* = -0.10) and CMJ (*r* = -0.16; both *P* > 0.05)
		UCMJ (10.6%)		No effect to SJ (*r* = 0.35) and CMJ (*r* = 0.22; both *P* > 0.05)
Philipp et al. [9]	Collegiate footballers (M; *n* = 24)	UCMJ (9.3 ± 6.5%)	Vertical Jump	↓ Vertical jump (*r* = -0.58, *P* < 0.05)
		UDJ (6.2 ± 6.5%)		No effect (*r* = -0.254, *P* > 0.05)
Teixeira et al. [31]	Amateur Crossfit (M; *n* = 13)	UCMJ (9.37 ± 5.66%)	SJ & CMJ	No effect to SJ (*r* = 0.26, *P* > 0.05) and CMJ (*r* = 0.14, *P* > 0.05)
	Amateur Crossfit (F; *n* = 11	UCMJ (9.51 ± 5.86%)	SJ & CMJ	No effect to SJ (*r* = 0.28, *P* > 0.05) and CMJ (*r* = 0.16, *P* > 0.05)
Torreblanca-Martínez et al. [32]	Elite soccer players (M; *n* = 19)	UCMJ (8.05 ± 6.77%)	CMJ & DJ	No effect to CMJ (*r* = -0.21) and DJ (*r* = -0.08; Both *P* > 0.05)
		UDJ (9.7 ± 5.41%)	CMJ & DJ	No effect to CMJ (*r* = -0.21) and DJ (*r* = -0.02; Both *P* > 0.05)

M = male; F = females; *n* = number of participants; UCMJ = unilateral countermovement jump; USJ = unilateral squat jump; UDJ = unilateral drop jump; (*x*%) = average asymmetry of participants as a percentage; ↓ = Decreased.

### 3.4. Change of direction performance

**Fig 4:**
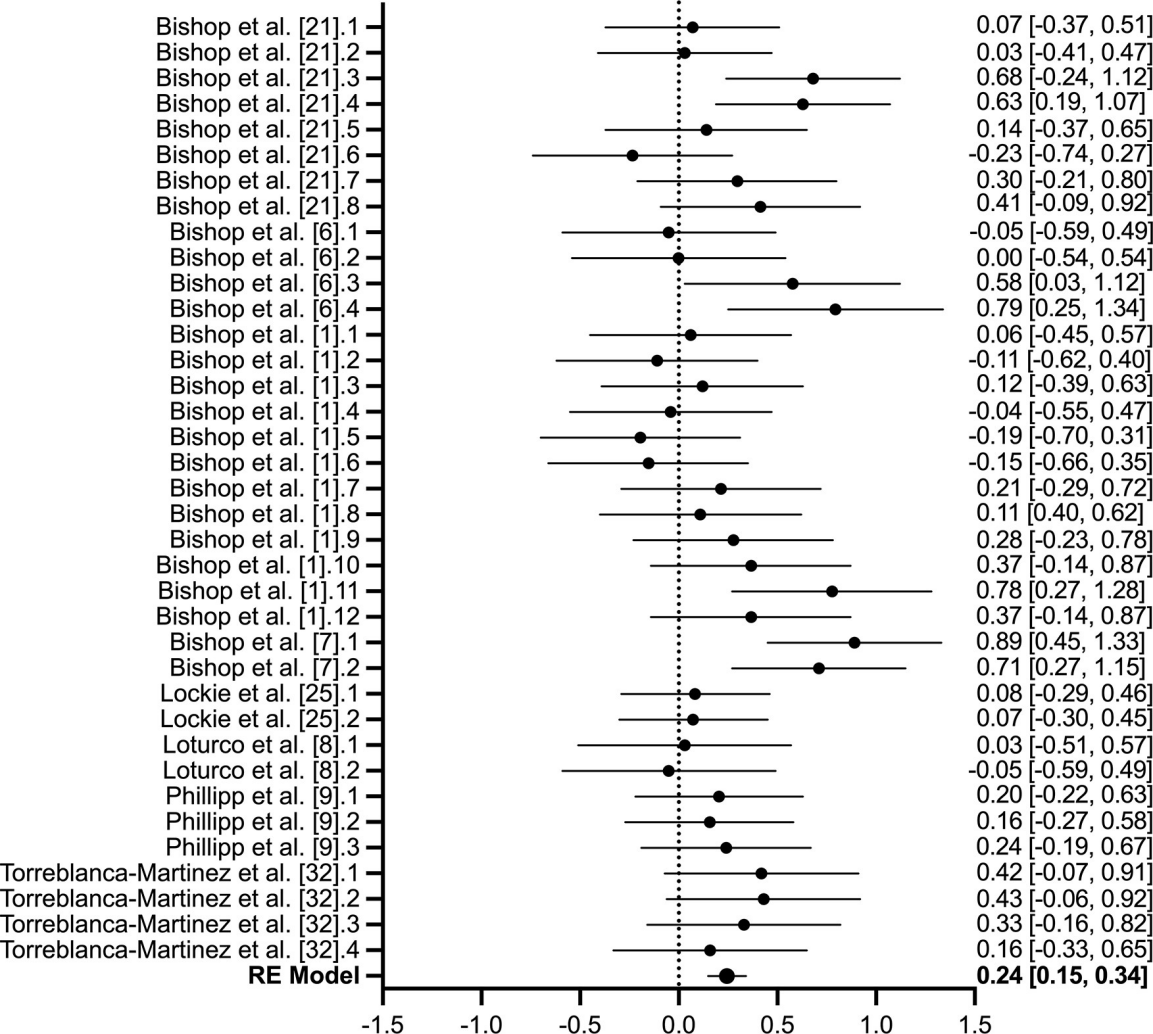
Forest plot of the 37-transformed effect sizes across eight studies (n = 187) assessing the effect of inter-limb asymmetries on change of direction performance via 505, zigzag, L-drill or pro-agility tests. The null line represents no correlation, with data presented to the left representing a negative correlation and to the right representing a positive correlation. For studies with repeated performance tests, a numerical order after the reference is used and aligns with the order presented in Table 2.

Eight studies (*n* = 187), of which 37-transformed effect sizes were provided, investigated the effect of inter-limb asymmetry on COD performance (Table 2). The quality of evidence assessing COD performance included five low quality studies and two very low-quality studies. No substantial or statistically significant heterogeneity was reported (*I*^*2*^ = 28.31%; Q-statistic, *P* = 0.051). The overall estimate effect was significant, though the magnitude was small (*Z*_*r*_ = 0.243, [95% CI: 0.150 to 0.337]; *r* = 0.24, [95% CI: 0.15 to 0.32]; *P* < 0.01; Fig 4). No significant bias was detected within eggers test (*P* = 0.478).

**Table 2:** Studies assessing the effect on lower, inter-limb asymmetries on change of direction performance (n = 187).

Study	Participants	Asymmetry Assessment (Average Asymmetry)	Performance	Findings
Bishop et al. [21]	Elite cricket players (M; *n* = 23)	UCMJ (9.57%)	505 COD (s)	No effect (R, *r* = 0.07; L, *r* = 0.03; Both *P* > 0.01)
		UDJ (11.49%)		↓ COD (R, *r =* 0.59; L, *r* = 0.56; Both *P* < 0.01)
	Elite soccer players (M; *n* = 18)	UCMJ (11.14%)		No effect (R, *r* = 0.14; L -0.23; Both *P* > 0.01)
		UDJ (6.51%)		No effect (R, *r* = 0.29; L -0.39; Both *P* > 0.01)
Bishop et al. [6]	Elite soccer players (F; *n* = 16)	UCMJ (9.3 ± 6.5%)	505 COD (s)	No effect (R, *r* = -0.05; L, *r* = 0; Both *P* > 0.05)
		UDJ (6.2 ± 6.5%)		No effect to COD-R (*r* = 0.52, *P* > 0.05). ↓ COD-L (*r* = 0.66, *P* < 0.05)
Bishop et al. [1]	Elite soccer players (M; *n* = 18)	UCMJ; pre-season (11.19 ± 9.58%)	505 COD (s)	No effect (R; *r* = 0.06; L, *r* = -0.11; Both *P* > 0.008)
		UDJ; Pre-season (8.42 ± 6.61%)		No effect (R; *r* = 0.12; L, *r* = -0.04; Both *P* > 0.008)
		UCMJ; mid-season (8.61 ± 6.99%)	505 COD (s)	No effect (R; *r* = -0.19; L, *r* = -0.15; Both *P* > 0.008)
		UDJ; mid-season (10.13 ± 9.15%)		No effect (R; *r* = 0.21; L, *r* = 0.11; Both *P* > 0.008)
		UCMJ; End-season (8.93 ± 6.83%)	505 COD (s)	No effect (R; *r* = 0.27; L, *r* = 0.35; Both *P* > 0.008)
		UDJ; End-season (10.42 ± 8.57)		↓ COD-R (*r* = 0.65, *P* < 0.008) No effect to COD-L (*r* = 0.35, *P* > 0.008)
Bishop et al. [7]	Elite soccer players (M; *n* = 23)	UCMJ (5.76%)	505 COD (s)	↓ COD (R, *r* = 0.71; L, *r* = 0.61; Both *P* < 0.01)
Lockie et al. [25]	Recreational team sport athletes (M; *n* = 30	UCMJ (10.4 ± 10.8%)	505 COD (s)	No effect (R, *r* = 0.08, *P* = 0.70; L, *r* = 0.07, *P* > 0.05)
Loturco et al. [8]	Elite soccer players (F; *n* = 16)	USJ (9.8%)	Zig-Zag COD test (m.s^-1^)	No effect (*r* = -0.03, *P* > 0.05)
		UCMJ (10.6%)		No Effect (*r* = 0.05, *P* > 0.05)
Philipp et al. [9]	Collegiate footballers (M; *n* = 24)	UCMJ (9.3 ± 6.5%)	Pro Agility Drill (s)	No effect (*r* = 0.20, *P* > 0.05)
		UDJ (6.2 ± 6.5%)	L-Drill (s) & Pro Agility Drill (s)	No effect (L-drill, *r* = 0.154, *P* > 0.05; Pro Agility drill, *r* = 0.236, *P* > 0.05)
Torreblanca-Martínez et al. [32]	Elite soccer players (M; *n* = 19)	UCMJ (8.05 ± 6.77%)	505 COD (s)	No effect (R, *r* = 0.401; L, *r* = 0.406: Both *P* > 0.05)
		UDJ (9.7 ± 5.41%)		No effect (R, *r* = 0.316; L, *r* = 0.158; Both *P* > 0.05)

M = male; F = females; *n* = number of participants; UCMJ = unilateral countermovement jump; USJ = unilateral squat jump; UDJ = unilateral drop jump; COD = change of direction; R = right leg; L = left leg; (*x*%) = average asymmetry of participants as a percentage; ↓ = Decreased.

### 3.5 Sprint performance

**Fig 5:**
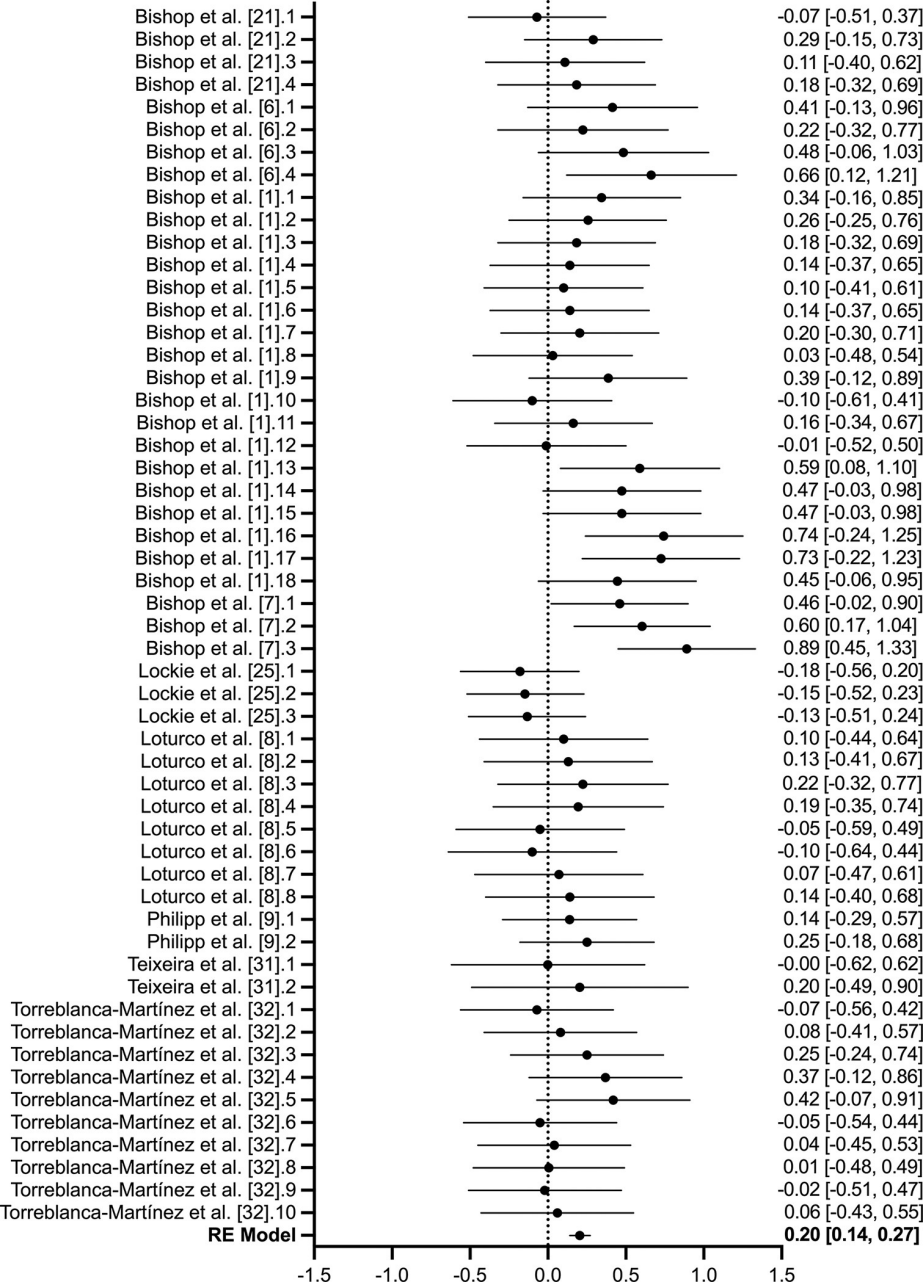
Forest plot of the 54-transformed effect sizes across nine studies (n = 211) assessing the effect of inter-limb asymmetries on sprint performance measured across 5 to 30-meters. The null line represents no correlation, with data presented to the left representing a negative correlation and to the right representing a positive correlation. For studies with repeated performance tests, a numerical order after the reference is used and aligns with the order presented in Table 3.

Nine studies (*n* = 211), of which 54-transformed effect sizes were provided, investigated the effect of inter-limb asymmetry on COD performance (Table 3). The quality of evidence assessing sprint performance included seven low quality studies and two very low-quality studies. No substantial or statistically significant heterogeneity was reported (*I*^*2*^ = 0%; Q-statistic, *P* = 0.343). The overall estimate effect was statistically significant though the magnitude was small (*Z*_*r*_ = 0.203, [95% CI’s: 0.136 to 0.270]; *r* = 0.2, [95% CI’s: 0.135–0.26]; *P* < 0.01; Fig 5). No significant bias was detected within Eggers test (*P* = 0.161).

**Table 3:** *Studies assessing the effect on lower*, *inter-limb asymmetries on sprint performance (*n *= 211)*.

Study	Participants	Asymmetry Assessment (Average Asymmetry)	Performance	Findings
Bishop et al. [21]	Elite Cricket players (M; *n* = 23)	UCMJ (9.57%)	10-m sprint (s)	No effect (*r* = -0.07, *P* > 0.008)
		UDJ (11.49%)		No effect (*r* = 0.28, *P* > 0.008)
	Elite Soccer players (M; *n* = 18)	UCMJ (11.14%)		No effect (*r* = 0.11, *P* > 0.008)
		UDJ (6.51%)		No effect (*r* = 0.18, *P* > 0.008)
Bishop et al. [6]	Elite soccer players (F; *n* = 16)	UCMJ (9.3 ± 6.5%)	10 & 30-m sprint (s)	No effect (10-m, *r* = 0.39; 30-m, *r* = 0.22; Both *P* > 0.05)
		UDJ (6.2 ± 6.5%)		No effect to 10-m sprint (*r* = 0.45, *P* > 0.05). ↓ 30-m sprint (*r* = 0.58, *P* < 0.01)
Bishop et al. [1]	Elite soccer players (M; *n* = 18)	UCMJ; pre-season (11.19 ± 9.58%)	5, 10 & 30-m sprint (s)	No effect (5-m, *r* = 0.33; 10-m, *r* = 0.25; 30-m, *r* = 0.18; All *P* > 0.008)
		UDJ; Pre-season (8.42 ± 6.61%)		No effect (5-m, *r* = 0.14; 10-m, *r* = 0.10; 30-m, *r* = 0.14; All *P* > 0.008)
		UCMJ; mid-season (8.61 ± 6.99%)	5, 10 & 30-m sprint (s)	No effect (5-m, *r* = 0.2; 10-m, *r* = 0.03; 30-m, *r* = 0.37; All *P* > 0.008)
		UDJ; mid-season (10.13 ± 9.15%)		No effect (5-m, *r* = -0.1.; 10-m, *r* = 0.16; 30-m, *r* = -0.01; All *P* > 0.008)
		UCMJ; End-season (8.93 ± 6.83%)	5, 10 & 30-m sprint (s)	No effect (5-m, *r* = 0.53; 10-m, *r* = 0.44; 30-m, *r* = 0.44; All *P* > 0.008)
		UDJ; End-season (10.42 ± 8.57)		↓ 5 & 10-m sprint (5-m, *r* = 0.63; 10-m, *r* = 0.62; *P* < 0.08). No effect to 30-m sprint (*r* = 0.42, *P* > 0.008)
Bishop et al. [7]	Elite soccer players (M; *n* = 23)	UCMJ (5.76%)	5, 10 & 20-m sprint (s)	No effect to 5-m sprint (*r* = 0.43, *P* > 0.05). ↓ 10 and 20-m sprint (10-m, *r* = 0.54, *P* < 0.05; 20-m, *r* = 0.71, *P* < 0.01)
Lockie et al. [25]	Team sport athletes (M; *n* = 30	UCMJ (10.4 ± 10.8%)	5, 10 & 20-m sprint (s)	No effect (5-m, *r* = -0.18, *P* > 0.05; 10-m, *r* = -0.15, *P* > 0.05; 20-m, *r* = -0.13, *P* > 0.05)
Loturco et al. [8]	Elite soccer players (F; *n* = 16)	USJ (9.8%)	5, 10, 20 & 30-m sprint (m.s^-1^)	No effect (5-m, *r* = -0.1; 10-m, *r* = -0.13; 20-m, *r* = -0.22; 30-m, *r* = -0.19; All *P* > 0.05)
		UCMJ (10.6%)		No effect (5-m, *r* = 0.05; 10-m, *r* = 0.1; 20-m, -0.07; 30-m, *r* = -0.14; All *P* > 0.05)
Philipp et al. [9]	Collegiate footballers (M; *n* = 24)	UCMJ (9.3 ± 6.5%)	40-y sprint (s)	No effect (*r* = 0.14, *P* > 0.05)
		UDJ (6.2 ± 6.5%)		No effect (*r* = 0.24, *P* > 0.05)
Teixeira et al. [31]	Amateur Crossfit (M; *n* = 13)	UCMJ (9.37 ± 5.66%)	20-m sprint (s)	No effect (*r* = -0.004, *P* > 0.05)
	Amateur Crossfit (F; *n* = 11	UCMJ (9.51 ± 5.86%)		No effect (*r* = 0.2, *P* > 0.05)
Torreblanca-Martínez et al. [32]	Elite soccer players (M; *n* = 19)	UCMJ (8.05 ± 6.77%)	10, 15, 20, 25 & 30-m sprint (s)	No effect (10-m, *r* = -0.07; 15-m, *r* = 0.08; 20-m, *r* = 0.25: 25-m, *r* = 0.35; 30-m, *r* = 0.40; All *P* > 0.05)
		UDJ (9.7 ± 5.41%)		No effect (10-m, *r* = -0.05; 15-m, *r* = 0.04; 20-m, *r* = 0.01: 25-m, *r* = -0.02; 30-m, *r* = 0.06; All *P* > 0.05)

M = male; F = females; *n* = number of participants; UCMJ = unilateral countermovement jump; USJ = unilateral squat jump; UDJ = unilateral drop jump; *x*-m = *x*-meter sprint; *x*-y = *x*-yard; sprint; (*x*%) = average asymmetry of participants as a percentage; ↓ = Decreased

## 4.0. Discussion

This systematic review and meta-analysis analysed the current literature to identify if an association between lower inter-limb asymmetries, quantified through unilateral jumps, on athletic performance exists. The findings of this review suggest that inter-limb asymmetries have no association with jump performance, however, present significant relationships with COD and sprint performance, albeit small. Although the quality of evidence within this area is considered low, the priori-quality rating (10.7/15) presented moderate quality and only two studies showed indirectness. However, emphasis is given on the inclusion of observational studies, thus the interpretation of these results should be done with caution.

The lack of association between asymmetry and jump performance, aligns with previous research [7, 8, 29–32], and supports computer simulations that compare symmetrical three-dimensional models against 10% asymmetrical models [33, 49]. Compensation from the unaffected limb could explain why asymmetries do not translate to a reduction in bilateral performances such as jumps. Computer simulations have found the dominant limb to exert greater force during the propulsive phase, compensating for the weaker limb to maintain performance [33, 49]. Furthermore, during the descending phase of a CMJ, the centre of mass has been reported to shift to the side of the dominant limb, allowing for this compensatory force production [33]. This suggests that individuals can alter movement mechanics to compensate for inter-limb asymmetries and maintain performance. However, interpreting these computer simulations should be taken with caution as inter-limb asymmetries were simulated as muscular strength imbalances as opposed to investigating unilateral jump asymmetries which have greater relevance to sport-specific movement patterns. Furthermore, the presence of heterogeneity within the jump performance analysis and the inclusion of observational research within this study causes potential imprecisions and invalidity of these results due to the lack of control over confounds and increased bias [46, 50]. Therefore, although no relationship exists between asymmetry and jump performance within this review, the certainty of a causal effect is lacking and must be considered when interpreting these findings.

Change of direction and sprint performance both had significant weak relationships with inter-limb asymmetry, indicating negative impacts to performance in the presence of asymmetry. These movements are more complex than jumping and, in addition to technique, rely heavily on reactive strength ability [51], the stretch shortening cycle [52], and eccentric-concentric strength [53]. Additionally, where jumping requires simultaneous use of both limbs (bilateral movement), COD and sprinting require independent use of each limb, making it more difficult for the non-affected limb to compensate for the affected limb. The results of this review support previous work, for example, Bishop et al. [1] reported significant differences in both COD and sprint performance between lower (3.22–4.61%) and higher asymmetry groups (13–18%), which suggest decremental effects of unilateral asymmetries with moderate to large effects (*d* = -0.96 to -1.40). However, the significant findings presented by Bishop et al. [1] were only identified on one occasion at the end of the season across a season long study (3-testing sessions); where it was concluded that asymmetry and performance were likely not related. Inconsistencies within the literature has previously been attributed to low percentages of asymmetries being investigated. For example Lockie et al. [25] proposed that no impact on performance was reported due to participants having < 15% asymmetries, suggesting that a larger deficit (> 15% asymmetries) is required to cause an impact on performance. Interestingly, the studies included within this review presented asymmetries below the 15% threshold (6–11%) which might account for the weak significant relationships reported between asymmetry and COD and sprint performance. Nevertheless, the findings of the current review extend those of Bishop et al. [2], where jumping based asymmetries were negatively associated with COD performance.

As previously stated, COD ability incorporates independent use of each limb. This unilateral independence may explain why COD tasks are able to detect asymmetry, thus have previously been employed as an approach to quantify asymmetry between the left and right limbs, further rationalising the results of this review, that inter-limb asymmetries negatively associate with COD performance [54]. However, there is a substantial variation in the degree of asymmetry found when comparing inter-limb asymmetries found via COD tasks and unilateral jumping [55]. This might result from the complexity of the COD task and the inclusion of both limbs (albeit individually) during the approach, deceleration, and acceleration phases, compared to unilateral jumping. However, this also supports the knowledge that asymmetry is task dependent, therefore, practitioners should attempt to use multiple asymmetry assessments to provide a more vigorous profile of athlete asymmetry [34].

### 4.1. Limitations and future research

Due to limited literature, sex and competition levels were pooled. As physiological- and sport-specific performance differs between men and women [56], and between playing standard levels [57–59], the ability to compensate for inter-limb asymmetries might vary. To note, although the participants sex was pooled, the ratio of men to woman participants was largely skewed towards men (men = 240; women = 43) and should be considered when interpreting the results of this study. Nevertheless, pooling such characteristics was appropriate to summarize the research field, as the limited transformed effects sizes that would be attained from a more specific eligibility criterion would increase the bias, losing control over type-1 error [45], and reduce the statistical power of the study [60]. Considering the limitations discussed within this review, it would be beneficial for future research to conduct sub-group analysis for all athletic variables (bilateral jumping, sprinting & COD) to provide greater specificity to practitioners. Additionally, as asymmetry cannot be manipulated to compare symmetry vs asymmetry within the same participant without completion of a longitudinal training intervention, the best proposed method to form a non-observational design would be to compare performance between individuals with low (< 15%) and high (> 15%) asymmetry, as done by Bishop et al. [1] and Lockie et al. [25]. The addition of kinematic and kinetic analysis would also allow for useful insight into the underpinning contributors of performance decrements or maintenance in the presence of asymmetry, as done in computer simulations.

## 4.2. Practical applications

Considering the implications that the ability to change direction and sprint have on sporting-performance and talent identification, this review suggests that inter-limb symmetry might provide small beneficial effects to an individual’s athletic performance. This could inform athletes and practitioners on the potential importance of asymmetry monitoring, specifically for performance tests underpinned by unilateral movements, such as COD and sprint performance. This small association found could have been due to the small asymmetries identified within the included studies therefore futures research should investigate populations where larger asymmetry dysfunctions are present. It is important for practitioners to comprehend the specific needs of each individual athlete and the magnitude of the individual’s asymmetry. Additionally, to achieve this, practitioners could regularly assess and monitor asymmetries to provide an insight into their athlete’s movement proficiencies and dysfunctions.

## 5.0. Conclusion

To conclude, the plausible effects of inter-limb asymmetries on athletic performance has recently been at the forefront of strength and conditioning research recently, however, an axiom conclusion has yet to be reached. Therefore, this study is the first systematic review with a meta-analysis to assess the association between inter-limb asymmetry and athletic performance. The data suggests that inter-limb asymmetries, quantified via unilateral jump performance, negatively impact COD and sprint, but not jump performance, in sporting cohorts.

## Supporting information

S1 Appendix Raw data retrieved from all included studies used for the vertical jump outcome meta-analysis. (CSV)

S2 Appendix Raw data retrieved from all included studies used for the change of direction outcome meta-analysis. (CSV)

S3 Appendix Raw data retrieved from all included studies used for the sprint outcome meta-analysis. (CSV)

S4 Appendix PRSIMA 2020 checklist. (DOCX)

S5 Appendix PRISMA 2020 abstract checklist. (DOCX)
